# Direct Characterization of the Relation between the Mechanical Response and Microstructure Evolution in Aluminum by Transmission Electron Microscopy In Situ Straining

**DOI:** 10.3390/ma14061431

**Published:** 2021-03-15

**Authors:** Seiichiro Ii, Takero Enami, Takahito Ohmura, Sadahiro Tsurekawa

**Affiliations:** 1 Research Center for Structural Materials, National Institute for Materials Science, Sengen 1-2-1, Tsukuba 305-0047, Japan; OHMURA.Takahito@nims.go.jp; 2 Department of Materials Science and Engineering, Graduate School of Science and Technology, Kumamoto University, Kurokami 2-39-1, Kumamoto 860-8555, Japan; 3 Department of Materials Science and Engineering, Graduate School of Engineering, Kyushu University, Motooka 744, Fukuoka 819-0395, Japan; 4 Elements Strategy Initiative for Structural Materials, Kyoto University, Yoshida-honmachi, Kyoto 606-8501, Japan; 5 Division of Materials Science and Chemistry, Faculty of Advanced Science and Technology, Kumamoto University, Kurokami 2-39-1, Kumamoto 860-8555, Japan

**Keywords:** transmission electron microscopy (TEM), in situ straining, indentation, dislocation, plastic deformation

## Abstract

Transmission electron microscopy in situ straining experiments of Al single crystals with different initial lattice defect densities have been performed. The as-focused ion beam (FIB)-processed pillar sample contained a high density of prismatic dislocation loops with the <111> Burgers vector, while the post-annealed specimen had an almost defect-free microstructure. In both specimens, plastic deformation occurred with repetitive stress drops (∆*σ*). The stress drops were accompanied by certain dislocation motions, suggesting the dislocation avalanche phenomenon. ∆*σ* for the as-FIB Al pillar sample was smaller than that for the post-annealed Al sample. This can be considered to be because of the interaction of gliding dislocations with immobile prismatic dislocation loops introduced by the FIB. The reloading process after stress reduction was dominated by elastic behavior because the slope of the load–displacement curve for reloading was close to the Young’s modulus of Al. Microplasticity was observed during the load-recovery process, suggesting that microyielding and a dislocation avalanche repeatedly occurred, leading to intermittent plasticity as an elementary step of macroplastic deformation.

## 1. Introduction

In situ observation by transmission electron microscopy (TEM) is a powerful technique to understand the dynamics of various reactions in materials under an external action, such as applied stress or high temperature [1,2]. By TEM in situ straining, many researchers have directly clarified the microstructural change during not only plastic deformation based on individual dislocation motion [3,4,5,6,7,8,9,10,11,12,13,14,15,16], but also the fracture behavior based on crack propagation [17,18,19,20]. TEM in situ straining has mainly been performed as a type of microscale tensile test using a conventional tensile holder [4] or by compression using a developed indentation holder [5]. In the 1960s, Fujita [6] succeeded in observing dislocation motion using a high-voltage electron microscope equipped with a tensile holder. Saka and Imura [7,8] further developed a tensile testing system for the transmission electron microscope and a TEM specimen preparation technique for tensile straining. Thereafter, TEM in situ straining became popular for clarification of the dislocation dynamics in various materials with face-centered cubic (fcc) [6,9,10,11], body-centered cubic (bcc) [12,13,14], and hexagonal close-packed structures [15,16] during plastic deformation at various temperatures. For brittle fracture, Ikuhara et al. [17] developed a TEM indentation holder working at elevated temperatures and reported the crack propagation by intra- and intergranular fracture in polycrystalline alumina at 1073 K. This indentation technique has been applied to several ceramic materials with brittle nature, such as magnesium oxide, silicon nitride, and sapphire (α-Al_2_O_3_) [18,19,20,21]. To clearly visualize the dislocations introduced during TEM in situ indentation experiments, Kondo et al. [22,23] recently used a double-tilt indentation holder and characterized an individual dislocation in a SrTiO_3_ single crystal and well-oriented SrTiO_3_ bicrystals with Σ5 and small-angle grain boundaries. They succeeded in observing individual dislocations and identified the activated slip system. They also found that SrTiO_3_ can be plastically deformed even at room temperature [22] and that the dislocation interaction with the grain boundary strongly depends on the grain boundary character [23].

Moreover, the development of the focused ion beam (FIB) system made it possible to prepare a TEM specimen containing a concerned local area, such as a grain boundary, with well-controlled shape and size for measuring the mechanical properties during in situ TEM experiments as well as the microstructural change. Warren et al. [5] applied the nanoindentation technique to FIB-processed specimens, and they accurately and simultaneously measured the stress by microstructural characterization. Minor et al. [24] observed the dislocation nucleation in a polycrystalline Al film by the TEM in situ indentation technique. They also found that the onset of dislocation motion occurred even before the increase in the applied force for the pop-in yielding phenomenon under theoretical shear stress [25]. Oh et al. [10] performed TEM in situ straining of an Al single crystal and investigated the strain-rate dependence of the deformation–microstructure relation. In their study, they observed the dislocation microstructure during the in situ tensile test and found that the dislocation density remained statistically constant during deformation at a strain rate of about 10^−4^ s^−1^. However, a sudden increase in the strain rate to 10^−3^ s^−1^ resulted in a considerable increase in the dislocation density, which indicates that deformation of a specimen with sub-micrometer size is strain-rate sensitive. In addition to dislocation nucleation at the incipient plasticity, Shan et al. [26] also performed TEM in situ compression of a Ni single-crystal pillar containing FIB-damage layers, and they observed the dislocation-free pillar after compression by TEM. They presumed that the dense dislocations were annihilated owing to the balance of the applied stress and image forces from the surface, which they called “mechanical annealing”. Zhang et al. [27] applied the TEM in situ indentation technique to a bcc Fe–3 mass% Si alloy single crystal. They found that the flow stress level depended on the character of the mobile dislocations; that is, the lower-stress stage was dominated by edge dislocations, and the higher-stress stage was governed by screw dislocations. They also investigated the correlation between the flow stress and the dislocation density during indentation and discussed the strain-softening based on the Johnston–Gilman model [28]. Very recently, Qu et al. succeeded in the development of an algorithm to automate the extraction of the pillar dimension and resulting in the calculation of the true stress-true strain (S–S) curve and strain hardening exponents [29]. As mentioned above, TEM in situ straining has provided new insights into not only the microstructural changes, such as individual dislocation motion, but also the quantitative mechanical properties. FIB sample preparation is a powerful tool for novel characterization. However, many lattice defects are unavoidably introduced during Ga^+^-ion irradiation [30], and they often cause difficulties in dislocation characterization. Moreover, they also cause extra strengthening during deformation. Kiener et al. [31] investigated the nanomechanics of Cu by an in situ tensile test in a transmission electron microscope. They used Cu specimens annealed in the transmission electron microscope after the FIB process and compared the deformation behavior with the as-FIB-processed sample. They found that the annealed specimens were always stronger than the as-FIB-processed specimens. Lee et al. [32] investigated annihilation of FIB-induced dislocations under in situ heating in a transmission electron microscope. They then investigated the deformation behavior and dislocation dynamics by scanning electron microscopy (SEM) in situ indentation and TEM in situ straining, respectively. However, to the authors’ knowledge, scarce information is available about the mechanical response associated with microstructural evolution. 

The main aim of this study is to investigate directly the microstructural evolution with the synchronized mechanical response through TEM in situ straining. The interaction between the dislocations and the internal defects with different initial density is discussed to understand the local mechanical behavior in sub-micrometer Al pillar samples. We also evaluate the quantitative relation between the dislocation motion and mechanical behavior.

## 2. Experimental Procedures

The material used in this study, which was supplied from Nippon Light Metal Co., (Shizuoka, Japan) was commercially available 99.99 mass% Al ingot. A plate with dimensions of 15 mm × 15 mm × 5 mm cut from the Al ingot was cold-rolled and annealed at 673 K for 12.6 ks in air to obtain a recrystallized microstructure and then furnace-cooled to room temperature. The average grain size was approximately 500 μm, and it was enough to prepare the single crystal sample for TEM in situ straining. 

The plates were mechanically ground and electrochemically polished to mirror surfaces. The microstructure and crystal orientation were characterized by SEM with a field-emission-type gun (Hitachi SU5000, Hitachi High-Tech Co., Hitachi, Japan) equipped with an electron backscattered diffraction system (EBSD) using TSL’s orientation imaging microscopy (OIM) software (OIM 7.3b, TSL solutions, Sagamihara, Japan).

The specimens for TEM in situ straining were picked up from a selected orientation region in the plate and fabricated into the pillar shape by a FIB–scanning electron microscope system (NB5000, HITACHI High-Tech Co., Hitachi, Japan). The size of the pillars was approximately 500 nm in width, 700 nm in length, and 300 nm in thickness. Although the specimens were relatively thick for conventional TEM observations, the thickness was kept at a few hundred nanometers to prevent buckling during compression straining.

The pillar-shaped specimens were categorized into two conditions. One was the post-annealed specimen after FIB processing. The other one was as-FIB processing (as-FIB). Post-annealed specimens were heated up to 523 K at a heating rate of 10 K/min and kept for 3 × 10^2^ s to reduce the FIB-induced defects to get the post-annealed sample. The abovementioned annealing was performed with a TEM in situ heating specimen holder in a vacuum atmosphere (JEM-2100 Plus. JEOL Co., Tokyo, Japan). The microstructural changes during heating were recorded step by step because the TEM system used for the in situ heating experiments was not equipped with a video capturing system.

TEM in situ straining was performed using a TEM indentation holder (Hysitron Picoindenter PI 95, Bruker Co., Minneapolis, MN, USA) and a JEOL JEM-2800 transmission electron microscope (Tokyo, Japan) operating at an acceleration voltage of 200 kV. The specimens were deformed by a flat-end-type tip with a diameter of 2.5 μm at a constant displacement rate of 1 nm/s in displacement-control mode at room temperature. Microstructure evolution during straining was recorded with a charge-coupled device camera (Orius SC200D, Gatan Co., Pleasanton, CA, USA) at a frame rate of 30 fps.

## 3. Results and Discussion

### 3.1. Annihilation of the FIB-Induced Defects during In Situ Heating

**Figure 1:**
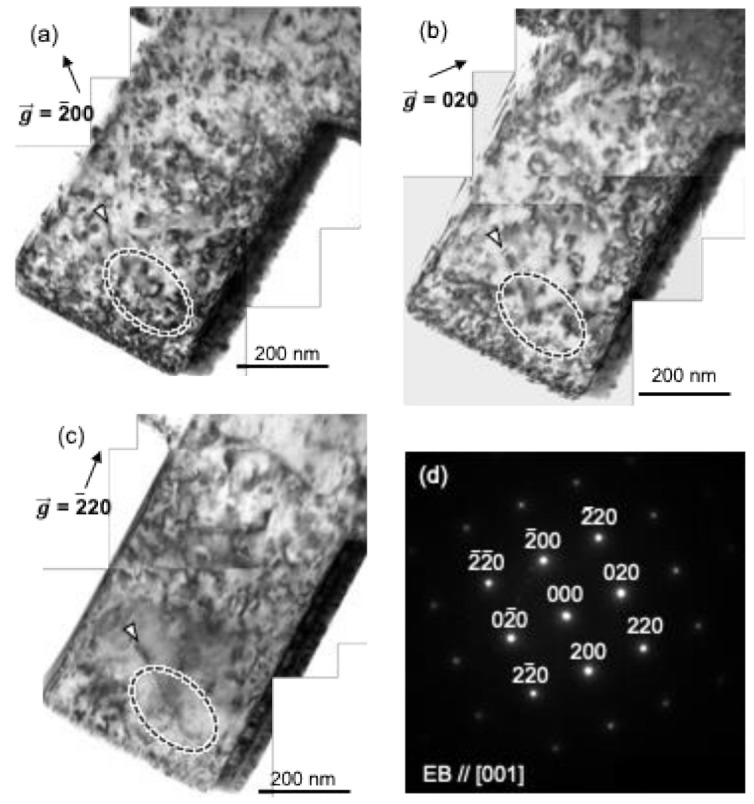
(**a**–**c**) Bright field images showing the defects induced by the focused ion beam (FIB) process in the as-FIB Al pillar. The images were taken under a near-two-beam condition with the diffraction vector $\vec{g}$, (**a**) $\vec{g}=\bar{2}00$, (**b**) $\vec{g}=020$, and (**c**) $\vec{g}=\bar{2}20$. (**d**) Selected-area electron diffraction pattern of the Al pillar shown in (**a**–**c**). The incident electron beam was parallel to [001]. Dashed ellipse and white arrows represent positions existing dislocation loop with $\vec{b}=1/3\;\langle 111\rangle$ and dislocation with $\vec{b}=1/2\;\langle 110\rangle$.

Bright-field images of the as-FIB Al pillar are shown in Figure 1a–c, and a selected electron diffraction pattern of the Al pillar is shown in Figure 1d. From the diffraction pattern, the incident electron beam was determined to be parallel to [001]. The images in Figure 1a–c were taken under the near-two-beam condition by excitation of the vectors $\vec{g}$ = $\bar{2}00$, 020, and $\bar{2}20$, respectively. The dark contrast along the right-hand side of all of the bright-field images corresponds to the side edge of the TEM pillar, because the specimens used in this experiment were thicker than those used for usual TEM observations. Many dot-like contrasts of dislocation loops are observed in Figure 1a–c, which were presumably induced by the FIB process. The Burgers vector ($\vec{b}$) of the dislocation loops can be determined by the invisible criteria of $\vec{g}\;\cdot \;\vec{b}=0$ under several two-beam conditions. Two types of Burgers vectors of the dislocation loops can be assumed in the specimen. The first is $\vec{b}$ = 1/2<110>, which is the most typical Burgers vector in fcc metals with the lowest self-energy introduced by plastic deformation. The second is $\vec{b}$ = 1/3<111>, which is frequently observed in fcc metals with a prismatic loop configuration introduced by ion irradiation [33]. By comparing the images in Figure 1a–c, it was found that some loop-shaped dislocation contrasts in the region indicated by the dashed ellipse are not visible in Figure 1c under the $\vec{g}=\bar{2}20$ condition, confirming that the Burgers vector of the dislocations is $\vec{b}$ = 1/3<111> of a prismatic dislocation loop. The other type of Burgers vector $\vec{b}$ = 1/2<110> is also observed as short line contrast being almost parallel to the longitudinal direction of the pillar indicated by the white arrowheads in Figure 1a,b, and it also disappeared under the invisible condition of $\vec{g}=\bar{2}20$ in Figure 1c. However, most of the defects in the specimen were determined to be <111>-type dislocation loops by several two-beam conditions.

**Figure 2:**
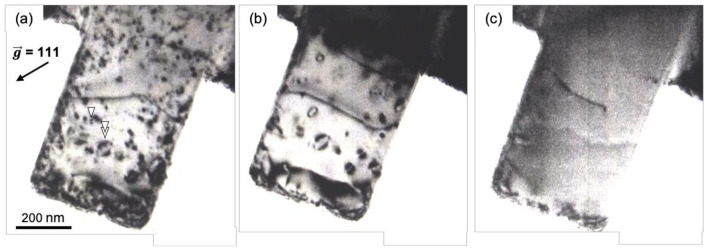
Series of snapshots showing the microstructural changes during the annihilation of the defects induced by FIB in Al in the TEM in situ heating experiment. (**a**) 303K, (**b**) 458 K, and (**c**) 573 K.

To investigate the effect of the dislocation density on the mechanical response during TEM in situ deformation, a TEM specimen was annealed by the TEM in situ heating technique to reduce the dislocation density. Snapshots of the dislocation structure that disappeared during heating are shown in Figure 2a–c. The relatively small dislocation loops indicated by the single arrowhead in Figure 2a started to shrink above 353 K and then disappeared during heating to 458 K (Figure 2b). Conversely, the relatively large dislocation loops indicated by the double arrowheads in Figure 2a remained at 458 K (Figure 2b), but they disappeared with further heating to 573 K (Figure 2c). It is presumed that the relatively small loops had larger line tension with a smaller curvature radius than the relatively large loops, resulting in shrinking. Conversely, the relatively large loops seemed to be enlarged owing to the migration mediated by vacancies during annealing and then escaped from the surface. In addition, by TEM–energy-dispersive spectroscopy analysis, it was experimentally confirmed that the Ga^+^ ions introduced by FIB fabrication almost completely escaped from the specimen during in situ heating [32]. We used the annealed specimen as the specimen with low dislocation density in the subsequent TEM in situ straining experiments.

### 3.2. TEM In Situ Straining of the as-FIB Sample with Relatively High Defect Density

**Figure 3:**
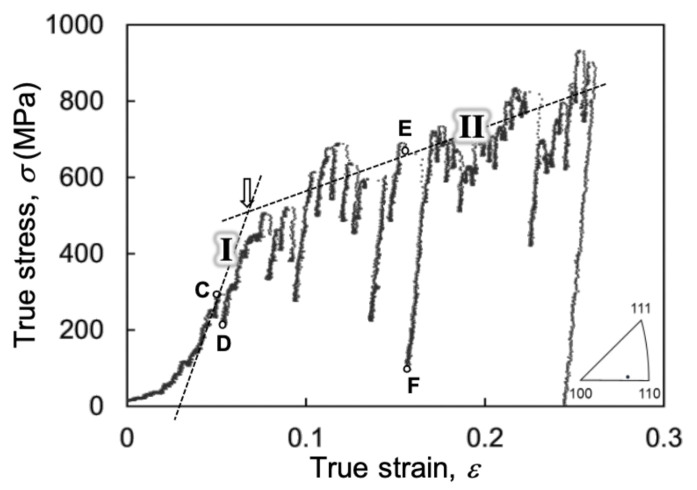
S-S curve of as-FIB Al pillar. Points C-F on the S-S curve correspond to snapshots in Figure 4c–f, respectively. The compression direction is shown by the stereographic triangle at the bottom right of the S–S curve.

The S-S curve of the as-FIB single-crystal Al pillar sample obtained by TEM in situ straining is shown in Figure 3. The compression axis was determined to be parallel to $\left[\bar{1}\;5\;12\right]$ by OIM analysis, as indicated by the standard triangle in the bottom right of the figure. Three specimens with the same orientation were tested, and no remarkable differences were observed for the specimens, indicating the repeatability of the mechanical behavior. In the S-S curve (Figure 3), there are many fluctuations in the load, and no clear yield point is observed. To estimate the yield stress, dashed linear lines were drawn along the S-S curves, and the curve was separated into the two regions of below (stage I) and above (stage II) about 0.07 strain (Figure 3), which appear to be analogous to elastic and elastoplastic deformation, respectively. The gradual slope is seen before stage I, which is due to a partial contact between the punch and sample with an unavoidable faultiness of the contact surface on the specimen and indenter. Therefore, the mechanical behavior before stage I was excluded to avoid uncertainties by the partial contact issue. Although stage I corresponds to the macroscopically elastic region, the S-S curve shows some fluctuations, but they appear to be less frequent and smaller in magnitude compared with stage II. Conversely, the S-S curve in stage II shows very frequent larger fluctuations. Because the stress fluctuation would result in a strain burst, the local peaks in the S-S curve can be presumed to be the initiation stress of the plastic strain burst. Therefore, in stage II, the dashed linear line determined by the least square method to the several peaks on the S-S curve is regarded as the flow stress. Accordingly, the pseudo-yield stress *σ_y_* can be defined as the intersection point between the two dashed linear lines (indicated by the arrow in Figure 3). Additionally, the flow stress at the strain of 0.1 (*σ*_0.1_) on the line can also be obtained. These stresses (*σ_y_* and *σ*_0.1_) were used for quantitative discussion of the deformation behavior in the following section. The average values of *σ_y_* and *σ*_0.1_ for the three tests with the same conditions were 421 and 454 MPa, respectively. Kunz et al. [34] investigated the mechanical behavior of a single-grained Al pillar by SEM indentation. They determined the relationship between the flow stress and the diameter of the cylindrical pillar through indentation. Although the sample shape with thin-plate pillars in this study was different from that in the previous study, the flow stresses were consistent for the same cross-sectional size. Based on these results, it the same tendency of the size effect was presumed even in the submicrometer-sized sample. However, since the size effect on the mechanical properties is out of the scope in our report, this shall be discussed in future work.

**Figure 4:**
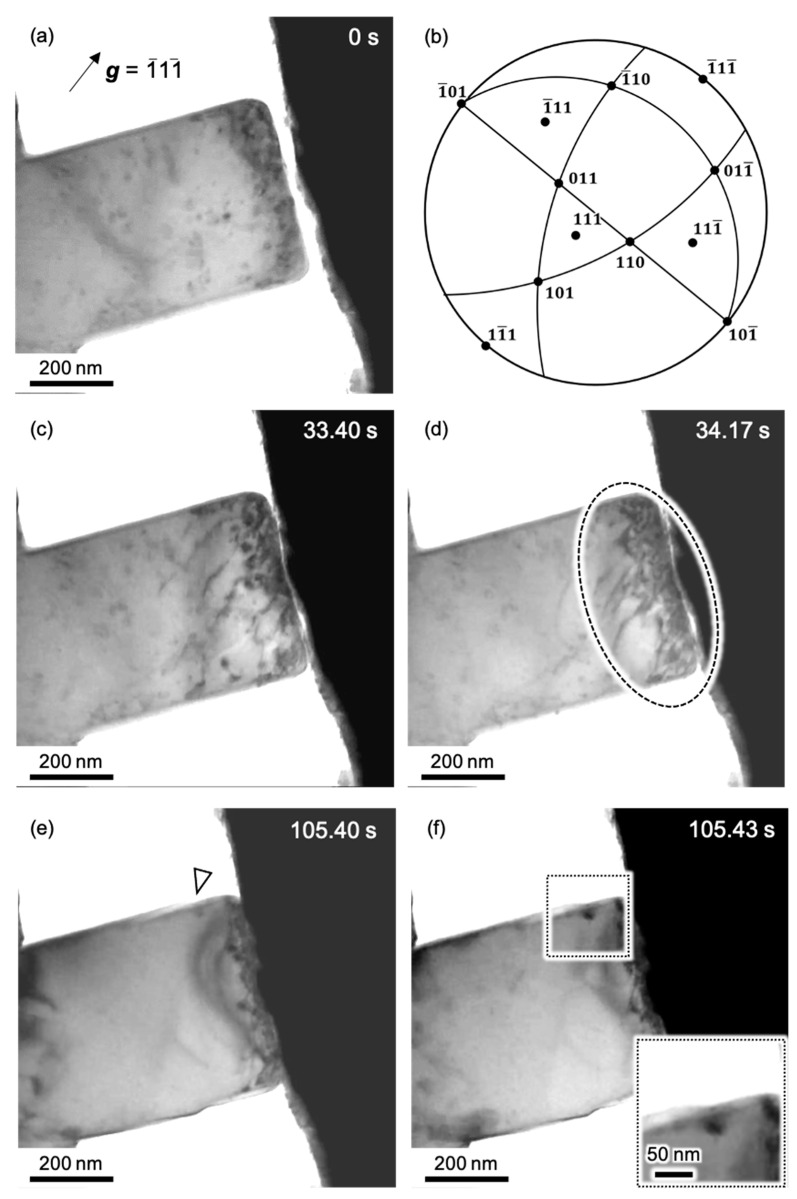
(**a**) Bright-field image of as-FIB Al pillar before compression. (**b**) Stereographic projection showing the geometry of the Al pillar in (**a**). (**c**–**f**) Series of snapshots during TEM in situ compression corresponding to points C-F on the S-S curve in Figure 3, respectively. The inset in the dotted square at the bottom right of (**f**) shows an enlarged image of the step structure. The region of the dotted square corresponds to the region indicated by a single arrowhead in (**e**).

The TEM bright-field image of the as-FIB specimen immediately before deformation is shown in Figure 4a, and the stereographic projection showing the geometry of the specimen in the TEM in situ specimen holder is shown in Figure 4b. Before compression, there were many dislocation loops in the specimen due to the FIB (Figure 4a). The dislocation structure is different from that in Figure 1 because the images in Figure 1 were taken using a conventional double-tilt holder to obtain a high contrast for the dislocation lines. Additionally, Figure 4a is under a different diffraction condition with limitation by a single-tilt operation in the in situ straining holder. A series of snapshots captured during TEM in situ compression of the as-FIB Al specimen is shown in Figure 4c–f. A video of this experiment is provided in the Supplementary Material (Video S1). Figure 4c,d correspond to the stress states immediately before and after the stress drop indicated by C and D on the S-S curve in Figure 3, respectively. The times of the snapshots are indicated at the top-right corners of the images. In Figure 4c, dislocations are observed near the top of the pillar. During a stress drop, the dislocation density increased in the region within the dashed ellipse shown in Figure 4d, which was 0.77 s after the snapshot shown in Figure 4c. This result demonstrates that the stress drop was caused by a collective dislocation glide within a very short period, like avalanche behavior, even though the stress drop from C to D in Figure 3 had a relatively small magnitude. After the pseudo-yield point, the magnitude of the stress drop was much larger than the event from C to D. For example, in another stress drop from E to F (Figure 3), the corresponding TEM images (Figure 4e,f) showed that a step (single arrowhead in Figure 4e) instantly grew (double arrowheads in the enlarged image of the dotted square in Figure 4f), suggesting remarkable plastic strain during the stress drop. Unfortunately, the dynamic motion of the dislocation was not captured during the event because of the out-of-diffraction condition used to obtain the high-contrast image of dislocation. This result also supports the model of the collective motion of dislocations for the stress drop [35].

### 3.3. Deformation Behavior of the Post-Annealed Al Pillar

**Figure 5:**
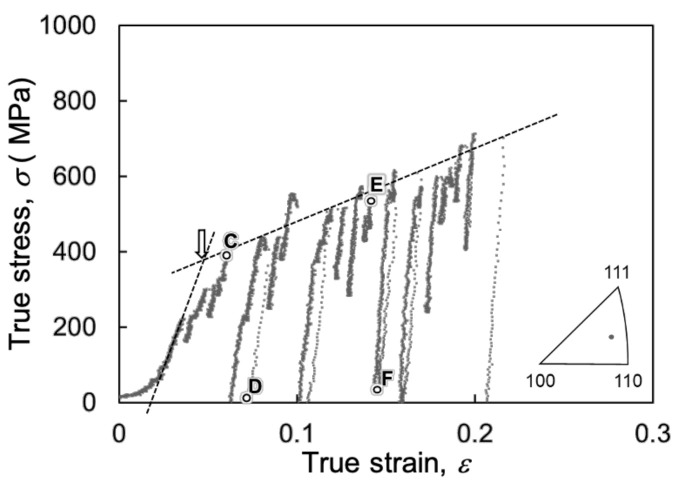
S-S curve of the post-annealed Al pillar. Points C-F on the S–S curve correspond to the snapshots in Figure 6c–f, respectively. The compression direction is shown by the stereographic triangle at the bottom right of the S-S curve.

The S–S curve obtained during TEM in situ compression of the post-annealed Al specimen is shown in Figure 5. The compression axis was $\left[13\;\bar{5}\;17\right]$, as indicated by the standard triangle at the bottom right of the figure. The two regions of elastic and elastoplastic behavior were approximately determined by the two dashed linear lines in the same way as for the as-FIB specimen in Figure 3. The *σ_y_* corresponding to the pseudo-yield stress defined as the intersect of the two linear lines indicated by the white arrow and *σ*_0.1_ corresponding to the flow stress at the strain of 0.1 values were determined to be 388 and 470 MPa, respectively. During the stress increase to *σ*_y_, small stress drops occurred a couple of times. After the pseudo-yield point, stress drops with a much larger magnitude were often detected, which was similar to the as-FIB sample (Figure 3). However, it should be noted that the stress was almost fully relaxed by reaching the horizontal axis with zero stress for some of the stress drops, which was not observed for the as-FIB sample. Because the post-annealed sample contained a much lower density of initial lattice defects, such as prismatic dislocation loops, the dislocations could more easily glide in the post-annealed sample than in the as-FIB sample. This is further discussed in the following section based on TEM characterization.

**Figure 6:**
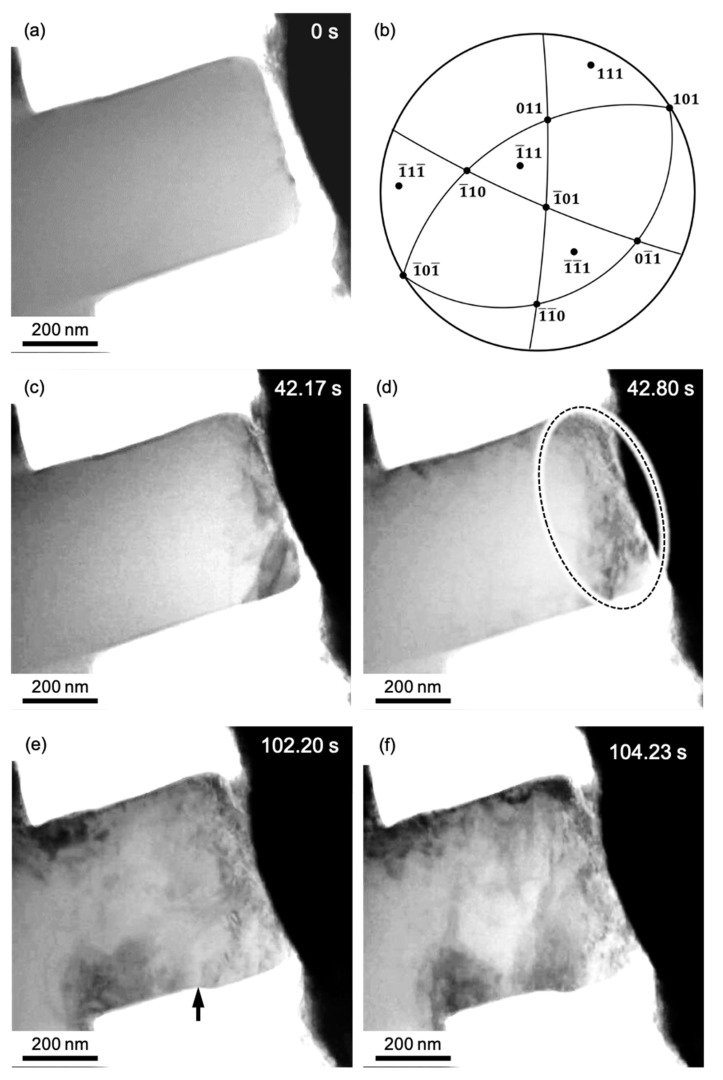
(**a**) Bright-field image of the post-annealed Al pillar before compression. (**b**) Stereographic projection showing the geometry of the Al pillar in (**a**). (**c**–**f**) Series of snapshots during the TEM in situ compression corresponding to points C-F on the S–S curve in Figure 5, respectively.

The TEM bright-field image of the post-annealed Al specimen immediately before deformation and the stereographic projection showing the geometry of the specimen in the TEM in situ holder are shown in Figure 6a,b, respectively. In Figure 6a, there is no contrast of defects, such as dislocation loops. A series of snapshots captured during TEM in situ compression of the post-annealed sample is shown in Figure 6c–f. A video of this process is also provided in the Supplementary Material (Video S2). Figure 6c,d correspond to the stress states immediately before and after the stress drop indicated by C to D on the S-S curve in Figure 5. The times are shown at the top-right corners of the images. In Figure 6c, the dislocation structure is observed close to the top of the pillar. During a stress drop, the dislocation density remarkably increased in the region within the dashed circle in Figure 6d, which was 0.63 s after that in Figure 6c. This was almost the same behavior as that of the as-FIB sample (Figure 4c,d). In the deformation stage after the pseudo-yield point, larger magnitude stress drops occurred, for example, the stress drop from E to F in Figure 5, corresponding to the images in Figure 6e,f. In Figure 6e, a step is observed on the pillar side, as indicated by the black arrow. From the stereographic projection in Figure 6b, it is expected that the slip system on $\left(\bar{1}1\bar{1}\right)$ should be activated for the formation of the step structure observed in Figure 6e,f. For the three possible Burgers vectors on $\left(\bar{1}1\bar{1}\right)$, $\left[\bar{1}\bar{1}0\right]$ leads to the highest Schmidt factor. Therefore, it is presumed that the slip system with $\left(\bar{1}1\bar{1}\right)\left[\bar{1}\bar{1}0\right]$ is preferably activated, although the $\left(\bar{1}1\bar{1}\right)\left[\bar{1}\bar{1}0\right]$ slip system corresponds to that with the third-highest Schmidt factor about the compression axis shown in Figure 5. After the stress drop, the step became larger at the same position, as shown in Figure 6f. It should be noted that the step size after the stress drop for the post-annealed sample in Figure 6f was much larger than that for the as-FIB sample in Figure 4f. This result suggests free-flight motion of a large number of dislocations in a limited area and then escape from the specimen surfaces for the post-annealed sample, which is consistent with the larger stress drop ∆*σ* in Figure 5. Lee et al. [32] also discussed the effect of FIB-induced dislocations on the deformation behavior. They individually investigated the mechanical behavior by ex situ microcompression and the microstructure change by in situ TEM. In their experiment, the annealed micropillar showed a stress drop in ex situ microcompression, and dislocations escaping from the sample surface were observed during deformation in in situ SEM and TEM, respectively. The results in our TEM in situ straining experiment were essentially the same as the previous phenomena, but the mechanical response and microstructural change, including the evolution of the dislocation structure, were simultaneously detected in this study, and it was found that the mechanical behavior such as an individual stress drop can be directly related to the dislocation motion.

### 3.4. Quantitative Comparison of the Mechanical Behavior

The S-S behavior showed a similar tendency for the two specimens (Figure 3 and Figure 5). In the early stage of deformation, the stress increased with a monotonic-like trend with less frequent and relatively small-magnitude stress drops. After the pseudo-yield stress, relatively large magnitude stress drops occurred with high frequency in both Al pillars. Furthermore, from the TEM in situ experiments, which are the combination of measurement of the mechanical response and microstructure observation, the relatively small stress drops resulted from a limited number of dislocation motions, while the relatively large stress drops were governed by the collective motion of many dislocations. The pseudo-yield stress *σ_y_* and flow stress at 0.1 strain *σ*_0.1_ values on the S-S curves of the as-FIB and post-annealed Al pillars are summarized in Table 1. These values are the average of five results under the same test conditions. The resolved shear stress (RSS) values for the slip system with the highest Schmidt factor at *σ_y_* and *σ*_0.1_ are also given in Table 1. The RSS at the yield point was higher for the as-FIB Al pillar than for the post-annealed Al pillar. The RSS at 0.1 strain showed the same trend. Therefore, higher stress was necessary for the dislocation glide motion in the as-FIB pillar because of the higher defect density induced by the FIB process. As shown in Figure 1, the as-FIB pillar contained many defects, such as prismatic dislocation loops and lattice dislocations. We also determined that the Burgers vector of these dislocation loops was 1/3<111>, which is immobile on any {111} plane in the fcc structure. When a mobile dislocation with the Burgers vector of 1/2<101> interacts with the <111> prismatic loop dislocation, a jog is formed on the <101> dislocation line to remarkably inhibit the glide motion. Hence, the mobile dislocations more often interacted with these defects during deformation for the as-FIB Al pillar than for the post-annealed Al pillar.

**Figure 7:**
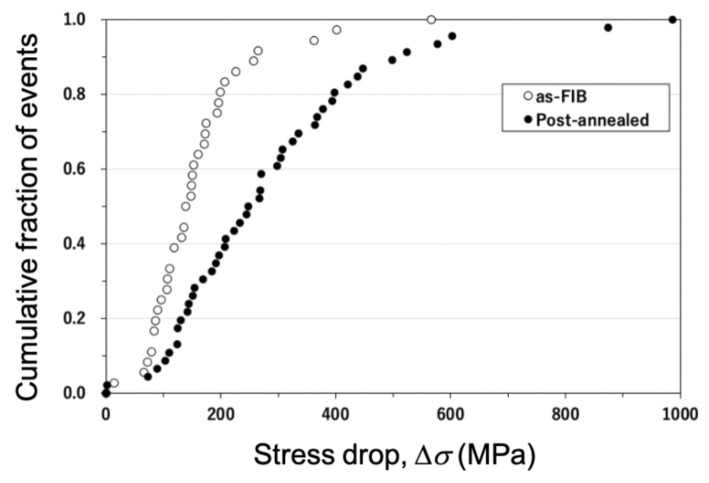
Cumulative distribution of the measured stress drop (∆σ) detected in the TEM in situ compression measurement. Open and solid circles represent the events detected in the as-FIB and post-annealed Al pillars, respectively.

The cumulative distributions of the measured stress drops (Δ*σ*) detected during TEM in situ straining of the as-FIB and post-annealed Al specimens are shown in Figure 7. The cumulative fraction of the stress drops steeply increased for the as-FIB Al sample (open circles). The trend for the post-annealed Al sample gradually changed in comparison with the as-FIB Al sample. The Δ*σ* value of the as-FIB Al sample at 50% cumulative fraction was 150 MPa, which was smaller than that of the post-annealed Al sample (275 MPa). Because the larger Δ*σ* value is caused by larger ∆*ε*, ∆*ε* can be converted into the shear strain ∆*γ*, which is given by the function ∆*γ* = *ρb*∆*x*. Hence, for the as-FIB Al sample, the smaller ∆*x* owing to interaction with the prismatic dislocation loops is presumably the main reason for the smaller ∆*σ*. This phenomenon is in good agreement with the previous report by Lee et al. [32].

**Figure 8:**
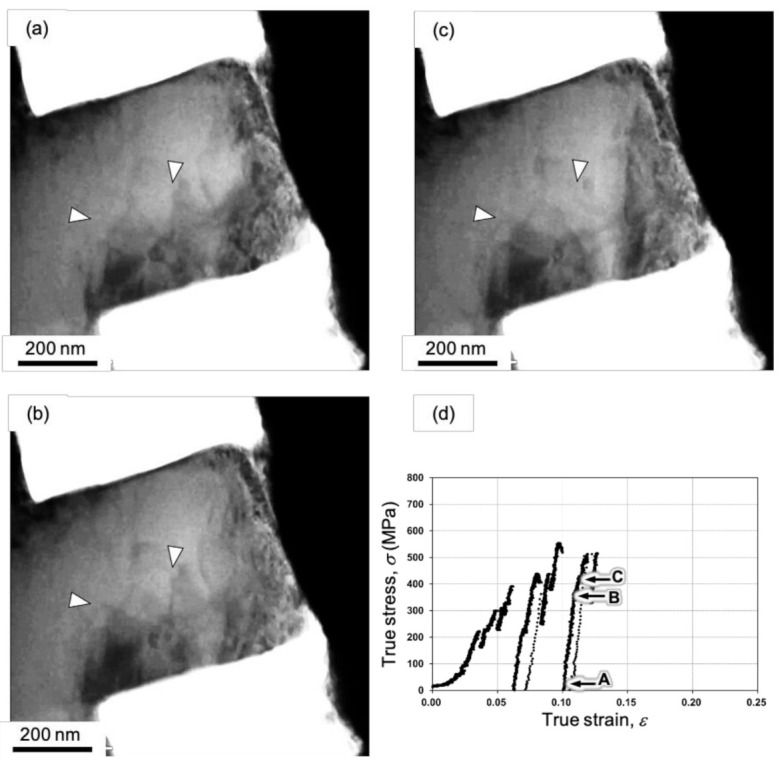
(**a**–**c**) Series of snapshots during the reloading process captured during TEM in situ compression of the post-annealed Al pillar. The images in (**a**–**c**) correspond to points A–C on the S–S curve in (**d**), respectively. At the points indicated by the arrowheads, the change in the contrast due to the motion of the dislocations are seen. This change is seen in the supplementary video (S3).

The deformation steps should be discussed on the basis of the results of TEM in situ straining, especially in the higher strain region after the pseudo-yield point. The stress responses after the stress drops in Figure 3 and Figure 6 were linear and steep, and the slopes ranged from 30 to 60 GPa, which are in the same range as the Young’s modulus of Al. This suggests that the deformation step during recovery of the load was mainly dominated by elastic deformation. Because the stress drop associated with the strain burst and reloading with elastic deformation was repeated on the S-S curve, the plastic strain evolution in the sample was intermittent plasticity [36]. Additionally, the reloading process was not purely elastic, but it included microplasticity in small magnitude. Snapshots of the recovery process captured during TEM in situ straining of the post-annealed Al sample are shown in Figure 8. The video of this process is provided in the Supplementary Material (Video S3). Figure 8a–c correspond to points A–C on the S-S curve in Figure 8d, respectively. Once the stress steeply dropped, slight strain reduction can be observed in the S–S curve. This is because of the inertial motion of the indenter, which cannot be naturally controlled by the feedback. The slope of this segment of the S-S curve is about 40 GPa, which is close to the Young’s modulus of Al, suggesting elasticity domination. Conversely, dislocation glide motion can be observed during the loading process from point A to C (Figure 8a–c). The arrowheads in Figure 8a–c indicate the same positions in the images. This dislocation motion indicates that deformation in this segment was not purely elastic deformation, but microplasticity also contributed to some extent. Note that even though some dislocations glided in the sample during the intermittent microplasticity, macrodeformation was still mainly governed by elastic deformation because the mobile dislocation density at the stress level was not sufficient.

A possible reason for the reloading process being macroscopically dominated by elastic deformation even though the dislocations were moving is as follows. TEM in situ straining was performed in displacement control mode with a constant nominal strain. 

When a certain strain is applied, the strain is balanced by a combination of plastic strain and elastic strain. The plastic strain is a function of the mobile dislocation density and the travel distance of the dislocation. If the dislocation density is not sufficient and the plastic strain cannot compensate for the given strain by the applied load, the elastic strain has to balance the strain, leading to a higher applied stress based on Hooke’s law. In particular, the dislocations easily escape from the surfaces of the specimen during TEM in situ straining, so it can also be considered to be a thin-film effect. When the applied stress reaches a critical value to activate plenty of dislocations and dislocation sources, another strain burst occurs to produce significant plastic strain. It should be noted that all of the dislocation motion cannot be captured because of the high velocity, suggesting flying motion in the post-annealed Al sample. Therefore, we can conclude that the macroplastic deformation in the present sample is dominated by intermittent plasticity consisting of microyielding and strain burst.

**Figure 9:**
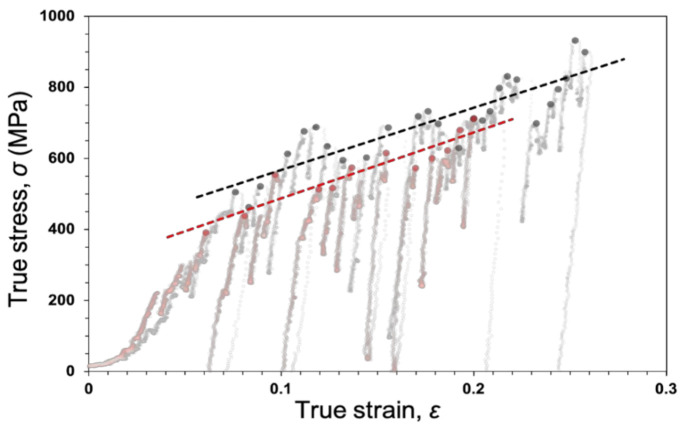
Plot of the stresses against the strain immediately before the stress drop, meaning the start point of the strain burst, for the as-FIB (black) and the post-annealed (red) Al pillars.

Another important point in the dislocation glide in each strain burst event is an elementary step of macroplastic deformation. Plots of the S-S data immediately before the stress drops, meaning the start points of the strain bursts, for the as-FIB and post-annealed Al samples, are shown in Figure 9. The complete S-S data also showed weak contrast marks. The dashed linear lines represent the traces of the plots on the same S–S curves as Figure 3 and Figure 5. For both samples, the stress values at the start points fluctuated. This behavior indicates that the critical stress to reactivate dislocation glide was dominated by a stochastic mechanism rather than a deterministic mechanism, because the stress values at the start points never decreased by a deterministic mechanism. The potential stochastic model is a thermally activated process in the dislocation motion by the Peierls stress [37]. Furthermore, the plot for the as-FIB Al pillar (black circles) fluctuated more than that for the post-annealed Al sample (red circles). This is confirmed by the correlation coefficients (*R*) of least square fitting to the dashed lines (*R* = 0.877 and 0.926 for the as-FIB and post-annealed Al samples, respectively). The smaller correlation coefficient for the as-FIB Al pillar compared with the post-annealed Al sample is presumably associated with the inhomogeneous microstructure, including the prismatic dislocation loops. As shown for the post-annealed sample in Figure 6, dislocations can glide out of the sample surface, and hence the dislocation density may not remarkably increase. For the as-FIB Al sample (Figure 4), the prismatic dislocation loops near the surface region prevent gliding dislocations from escaping from the sample surface, resulting in higher density and inhomogeneous dislocation structures. In the inhomogeneous dislocation structures, the distribution of the critical stress is suitable for activating a dislocation glide, leading to the fluctuation of the initial stress with a lower *R* value. The dislocation structure and associated distribution of the critical stress changed with strain evolution, and the dislocation with the lowest critical stress preferentially started to glide, so the average of the critical stress distribution increased. The dashed lines in Figure 9 show monotonically increasing trends, suggesting that the average stress of the distribution increased with strain as strain hardened.

## 4. Conclusions

TEM in situ straining measurements, which make it possible to reveal the mechanical response associated with microstructure changes, have been performed for Al single-crystal pillars with different initial defect densities. We succeeded in linking the elemental mechanical behavior and microstructure evolution directly through the in situ measurements. The effect of lattice defects, which were introduced by the FIB process, on the mechanical behavior was also investigated and compared with the defect-free post-annealed Al pillar. The results are summarized as follows.

1. In the as-FIB Al sample, a high-density of lattice defects was observed with a conventional transmission electron microscope. Most of the defects introduced by FIB fabrication were prismatic dislocation loops with the Burgers vector 1/3<111>. TEM in situ annealing at 523 K significantly reduced the number of lattice defects introduced by FIB fabrication.
2. The S–S curves obtained during TEM in situ straining of the as-FIB and post-annealed Al samples showed repetitive stress drops. The stress drops frequently occurred, especially after the pseudo-yield point. The magnitude of the stress drops was smaller for the as-FIB Al sample than for the post-annealed Al sample. In addition, the stress in the whole range of the S-S curve was higher for the as-FIB Al sample than for the post-annealed Al sample.
3. The pseudo-yield point, *σ*_y_, of the as-FIB and the post-annealed Al samples was 421 MPa and 328 MPa, respectively and the *σ*_y_ of the as-FIB was approximately 20% higher than that of the post-annealed one.
4. The TEM in situ straining experiments revealed that the stress drops on the S–S curve were accompanied by collective dislocation motions. The change in the dislocation density and the traveling distance of the dislocations strongly affected the magnitude of the stress drop.
5. The cumulative fraction of the measured stress drop detected in the as-FIB Al samples were more steeply increased in comparison to the post-annealed samples. The stress drop at 50% cumulative strain was 150 MPa in the as-FIB Al sample and 275 MPa in the post-annealed one, which is almost twice higher than that in the post-annealed sample. This means that the dislocation loops in the as-FIB act as an obstacle for the mobile dislocations.
6. After the stress rapidly decreased, the stress increased to the original stress state within 1 min with mainly elastic behavior, which is identified as intermittent plasticity. However, because microplasticity also occurred during recovery of the stress, the stress values for starting the strain bursts were not consistent, and they significantly fluctuated. The intermittent plasticity is triggered by a thermally activated process.

## Figures and Tables

**Table 1:** Mechanical properties of the Al pillar obtained by TEM in situ compression measurement. The stresses (*σ*_y_ and *σ*_0.1_) were measured from the S–S curves. The resolved shear stresses (*τ*_y_ and *τ*_0.1_) were evaluated by the slip system activating during the TEM in situ experiments. The activating slip system has the highest Schmidt factors (*m*), namely 0.4988 (as-FIB) and 0.4634 (Post-annealed).

Al Piller	Pseudo-Yield Stress, *σ*_y_	Flow Stress at the Strain of 0.1, *σ*_0.1_
as-FIB	421 MPa	534 MPa
Post-annealed	328 MPa	505 MPa
–	*τ*_y_ (= *m σ*_y_)	*τ*_0.1_ (= *m σ*_y_)
as-FIB	210 MPa	266 MPa
Post-annealed	152 MPa	234 MPa

## Data Availability

Data is contained within the article.

## Supplementary Materials

The following are available online at https://www.mdpi.com/1996-1944/14/6/1431/s1, Video S1: The microstructure evolution recorded by the TEM in situ compression experiment of the as-FIB Al specimen. The S-S curve obtained simultaneously is synchronized with the microstructure evolution. Video S2: The microstructure evolution recorded by the TEM in situ compression experiment of the post-annealed Al specimen. The S-S curve obtained simultaneously is also synchronized. Video S3: The microstructure evolution during the reloading process after stress drop in the post-annealed Al pillar. Text S4: Brief interpretation of the videos of S1–S3.
