# Supplementary material for: Direct Characterization of the Relation between the Mechanical Response and Microstructure Evolution in Aluminum by Transmission Electron Microscopy In Situ Straining
Source: Materials (Basel). 2021 Mar 15;14(6):1431. doi: 10.3390/ma14061431 (PMC7998695; doi:10.3390/ma14061431)
Supplement: Supplementary file 1 [file materials-14-01431-s001.zip › Supplementary - FINAL VERSION/Supplementary.pdf]

materials

Supplementary Materials

Direct Evaluation of the Relation between the Mechanical Response and Microstructure Evolution in Aluminum by Transmission Electron Microscopy In Situ Straining

Seiichiro Ii [1,*], Takero Enami [2], Takahito Ohmura [1,3,4] and Sadahiro Tsurekawa [5,*]

1 Research Center for Structural Materials, National Institute for Materials Science, Sengen 1-2-1, Tsukuba 305-0047, Japan; OHMURA.Takahito@nims.go.jp
2 Department of Materials Science and Engineering, Graduate School of Science and Technology, Kumamoto University, Kurokami 2-39-1, Kumamoto 860-8555, Japan
3 Department of Materials Science and Engineering, Graduate School of Engineering, Kyushu University, Motooka 744, Fukuoka 819-0395, Japan
4 Elements Strategy Initiative for Structural Materials, Kyoto University, Yoshida-honmachi, Kyoto 606-8501, Japan
5 Division of Materials Science and Chemistry, Faculty of Advanced Science and Technology, Kumamoto University, Kurokami 2-39-1, Kumamoto 860-8555, Japan
* Correspondence: II.Seiichiro@nims.go.jp (S.I.); turekawa@kumamoto-u.ac.jp (S.T.); Tel.: +81-29-859-2160 (S.I.)

Citation: Ii, S.; Enami, T.; Ohmura, T.; Tsurekawa, S. Direct Characterization of the Relation between the Mechanical Response and Microstructure Evolution in Aluminum by Transmission Electron Microscopy In Situ Straining. *Materials* **2021**, *14*, 1431. https://doi.org/10.3390/ma14061431

Academic Editor: Oleg Lebedev

Received: 12 February 2021
Accepted: 10 March 2021
Published: 15 March 2021

Publisher's Note: MDPI stays neutral with regard to jurisdictional claims in published maps and institutional affiliations.

Copyright: © 2021 by the authors. Licensee MDPI, Basel, Switzerland. This article is an open access article distributed under the terms and conditions of the Creative Commons Attribution (CC BY) license (http://creativecommons.org/licenses/by/4.0/).

As supplementary materials, movies recorded by the TEM in situ compression experiments can be seen.

Video S1 is the movie of the microstructure evolution recorded by TEM in situ compression of the as-FIB Al specimen. The stress-strain (S-S) curve obtained simultaneously by this in situ experiment is synchronized with the microstructure evolution. The playback speed of the video is five times faster than the original speed in the in situ compression experiment. At the beginning of the movie (2 sec in the video), the dislocation starts to nucleate from the top of the pillar. Around the 6 sec corresponding to the points C and D in Figure 3 and Figure 4c and Figure 4d in the main text, stress drop is detected in the S-S curve, and in the TEM image, dislocation density obviously increases during the stress drop. Further compression, the deformation progressed with the repeated stress drop. However, even at the late stage of the deformation, few dislocation contrasts become to be seen. That is considered to be due to the change in the diffraction condition during the deformation. On the other hand, the step can be formed at the top-sidewall in that stage. The detail is described in the Section 3.2.

Video S2 is the movie of the microstructure evolution recorded by the TEM in situ compression of the post-annealed Al specimen, showing the microstructure change and the synchronized S-S curve as well as the video S1. The playback speed of the video S2 is also five times faster than the original speed. Even though contrast is weak, at the time of 5 sec, dislocation contrast can be seen at the top of the pillar with the first stress drop in the S-S curve. Further deformation, dislocation is continuing to nucleate around the top of the pillar. At 8 sec corresponding to point C and D in Figure 5 and Figure 6c and Figure 6d, the contrast is instantly changed during the stress drop, and the dislocation at not only the top but also both sidewalls can be seen. And then, the contrast changes during the plural stress drop, and the step at the bottom-sidewall is seen at the late stage of the deformation. This is also described in the Section 3.3.

Video S3 shows the microstructure evolution during the reloading process after stress drop. The snapshots in Figure 8 are captured from this time interval. Note that the dislocation contrast is moving from right to left at the rightward arrowhead and the center

to bottom at the downward one in Figure 8a–c around 5 sec. That means dislocation activates even at the reloading process, which is apparently elastic behavior. Details are explained in the Section 3.4.
